# Knowledge and Practice of Clinicians regarding Syndromic Management of Sexually Transmitted Infections in Public Health Facilities of Gamo Gofa Zone, South Ethiopia

**DOI:** 10.1155/2015/310409

**Published:** 2015-10-29

**Authors:** Addisu Alemayehu, Wanzahun Godana

**Affiliations:** ^1^Department of Nursing, College of Medicine and Health Sciences, Arba Minch University, P.O. Box 21, Arba Minch, Ethiopia; ^2^Department of Public Health, College of Medicine and Health Sciences, Arba Minch University, P.O. Box 21, Arba Minch, Ethiopia

## Abstract

*Background*. Sexually Transmitted Infections (STIs) are the leading causes of morbidity among young adults. This study assessed the knowledge and practice of clinicians regarding syndromic management of STIs in public health facilities of Gamo Gofa Zone, Southern Ethiopia.* Methods*. Facility based cross-sectional study with mixed methods of data collection was conducted in public health facilities of Gamo Gofa Zone. The study included 250 clinicians and 12 health facilities, 26 mystery clients were hired, and 120 STI patient cards were reviewed. Data was entered in EPI info version 7.0.1 and analyzed by SPSS version 20.* Results*. Of the participated clinicians, 32 (12.8%) were trained on syndromic management of STIs. Highest knowledge of clinicians was for urethral discharge (27.2%). Professional category of clinicians and type of health facility (AOR = 0.194; 95% CI = 0.092, 0.412) were determinants of urethral discharge knowledge. Of the cards reviewed, only in 8.3% of cards and 19.23% of mystery clients did the clinicians correctly follow the guideline.* Conclusion*. Knowledge and practice of clinicians regarding syndromic management of STIs in study area were poor. Efforts should be made to increase the knowledge of clinicians by providing training on syndromic management of STIs and supportive supervision should be regular.

## 1. Introduction

Sexually transmitted infections (STIs) are infections that are mainly transmitted from person to person through sexual contact. The causative agents include bacteria, viruses including human immunodeficiency virus (HIV), and parasites [1, 2]. STIs are important because of their magnitude, potential complications, and their interaction with HIV/AIDS. The effect of STIs was significant in affecting reproductive potential of affected individuals. STIs and their associated complications are regarded as one of the top five reasons for which adults avail health care services in developing countries. Misdiagnosis and management have long lasting complications [3–6]. The magnitude of STIs is being increased from time to time and from place to place [1, 4, 7]. The objective of syndromic approach for STI management is to identify and treat a syndrome with combination therapy as well as education of the patient, condom supply, counseling, notification, and management of sexual partners and HIV counseling and testing (HCT). It was recommended for resource limited setting where both laboratory facilities and skilled personnel were unavailable. It is widely practiced in Ethiopia since 2001 [4, 8–10].

Infertility, congenital abnormality, adverse neurological condition, cardiovascular risks, ectopic pregnancy, anogenital cancer, and even premature death of neonates were some of the complications of misdiagnosed and mismanaged STIs [11, 12].

Knowledge and practice of health professional involved in care and support of STI patients determine its outcome. But, studies conducted in developing countries indicate that the knowledge and practice of clinicians regarding syndromic management of STIs were insufficient. In study conducted in Karachi, Pakistan, and Windhoek, Namibia, the knowledge of health workers regarding syndromic management of STIs was insufficient [12, 13]. And study conducted in Pakistan and six countries in West Africa shows that the practice was insufficient [14, 15]. Similarly the study conducted in Kathmandu, Nepal, also shows that knowledge and practice regarding STI/HIV have not been found sufficient among health workers [16, 17].

In Ethiopia, there are no studies addressing the knowledge and practice of clinicians regarding syndromic management of sexually transmitted infections. Therefore, this study was designed to assess the knowledge and practice of clinicians concerning syndromic management of STIs in public health facilities of Gamo Gofa Zone, Southern Ethiopia.

## 2. Methods and Materials

### 2.1. Study Setting

The study was conducted in public health institutions of Gamo Gofa Zone which is located 505 km south west from Addis Ababa, the capital city of Ethiopia, and 275 km from Hawassa, the capital of the South Nations Nationalities and People Regional State (SNNPRS). According to the 2013 zonal health department report the population of the zone was 1,960,417 (960,604.33 were males and 999,812.67 females). Gamo Gofa Zone has 15 woredas and two city administrations. There are 3 hospitals and 68 health centers offering health care services for the population in the zone. The number of physicians including specialists was 40 and all category health professionals are 1495. There are 958 health extension workers including urban health extension workers. Facility based mixed method study design with both quantitative and qualitative methods of data collection was conducted from January 1 to February 28, 2015.

### 2.2. Study Population

All health professionals working in public health facilities in Gamo Gofa Zone and all STI patients in Gamo Gofa Zone were source population. All health professionals working in these facilities which treat STI patients and STI patients in catchment area were study population. All health professionals working as clinician and health professionals working as managers in the selected health facility were included.

### 2.3. Sample Size and Sampling Techniques

Sample size was calculated by using stat calc program of EPI Info by using the following assumption: 18% (proportion of knowledge of health care provider about syndromic management of STIs in Pakistan) [18], 95% confidence level, and 5% level of precision. So a total of 227 was calculated then by adding 10% nonresponses rate, and the final sample size was 250 clinicians. For mystery clients, a total of 26 clients were hired. And for observation, 120 STI patient cards (10 in each health facility) were reviewed.

Two hospitals and 10 health centers were selected by simple random sampling technique and sample size was proportionally allocated to those study health institutions. Then by using cluster sampling, all eligible health professionals in the selected health institution were selected. The cluster is based on the case team of respective health institutions. For mystery client interview, three clients with different syndromes in each hospital and two in each health center were used.

### 2.4. Data Collection Instrument and Procedures

Data was collected using questionnaire adopted from District STI Quality of Care Assessment (DISCA) tool [19, 20] and for specific knowledge of syndromes self-administered questionnaire was used. Cards of STI patients were observed and reviewed. Tools adapted from DISCA or semistructured questionnaire was used for mystery clients.

### 2.5. Variables

#### 2.5.1. Dependent Variables

Knowledge and practice of clinicians were the dependent variables.

#### 2.5.2. Independent Variables

Age of clinicians, sex of clinicians, year of work, profession, training status, types of health, and facility were the independent variables.

### 2.6. Operational Definitions

1. *Knowledge of clinicians* is measured by how correctly the clinician responded to the knowledge questions according to the national guideline that is correct drug, correct dose, correct frequency, and correct duration.
2. *Practice of clinicians* is measured by how correctly the clinician followed the national guideline to treat STI patient in the past and currently to treat the mystery client that is correct diagnosis, correct drug, correct dose, correct frequency, and correct duration.

### 2.7. Data Quality Assurance

The data collectors were trained for two days on how to act as real patient and collect data. The investigators have checked data completeness, clarity, and consistency. Before analysis the data were cleaned thoroughly to check for errors during entry.

### 2.8. Data Analysis

Each completed questionnaire was checked manually for completeness before data entry. The data were coded and entered into EPI Info version 7.0.1 and cleanup was made to check accuracy and consistency. Finally, data was exported to SPSS version 20 for further cleaning and analysis. Descriptive statistics and bivariate and multivariate analysis were done using binary logistic regressions. Statistical significance was set at *P* ≤ 0.05 and adjusted odds ratio was computed to assess the association between explanatory and outcome variables.

### 2.9. Ethical Considerations

Ethical clearance was obtained from ethical review committee of College of Medicine and Health Sciences, Arba Minch University. Permission to conduct the study was also sought from the administrative authority of the respective health institution. Before enrolling any of the eligible study participants, informed written as well as verbal consent was taken. Information was provided for the purpose of the study, and benefits and the confidential nature of the study were described and discussed for each participant.

## 3. Results

### 3.1. Sociodemographic Characteristics

A total of 26 mystery clients were interviewed, 250 clinicians responded to self-administered questionnaire, and 120 cards of STI patients were reviewed.

The overall response rate was 100%. Regarding clinicians, 174 (69.6%) were diploma nurses, 142 (56.8%) were male, and more than half of the clinicians, 141 (56.4%), were below the age of 30 years and only quite a small proportion, 15 (6%), were at the age of 50 and above. The mean age of the clinicians was 31.43. Out of all, 50% of the clinicians have a median of six years of experience (Table 1). B.S. nurses are the most experienced group with a mean of 12.6 years of experience. Diploma nurses, health officers, and medical doctors have an average of 8.49, 7.73, and 2.67 years of experience, respectively.

**Figure 1:**
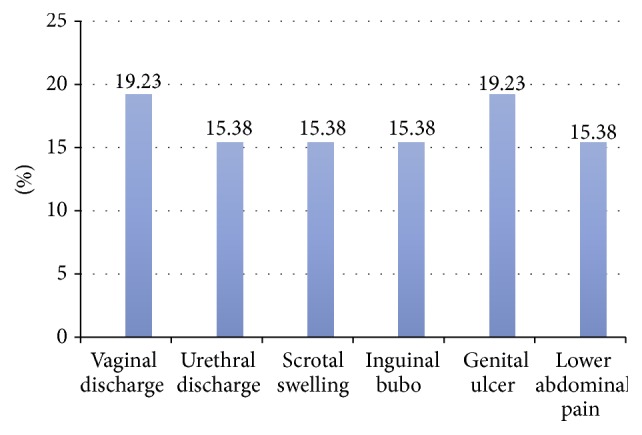
Syndromes mystery clients treated for in public health facilities of Gamo Gofa Zone, Southern Ethiopia, 2015.

Concerning mystery clients, the clients were 5 with vaginal discharge, 4 with urethral discharge, 4 with scrotal swelling, 4 with inguinal bubo, 5 with genital ulcer, and 4 with lower abdominal pain (Figure 1). From the total of 26 mystery clients, 13 were males and 13 were females.

### 3.2. Training of Clinicians on Syndromic Management of STIs

Of the clinicians participating in the study, 32 (12.8%) were trained on syndromic management of STIs and 50% of these had received their training before a median of 4.5 years prior to this study. The clinicians were trained 1 to 11 years prior to the study, with a mean duration of 4.88 years.

### 3.3. Knowledge of Clinicians

Clinicians who correctly mention the name of the drugs for urethral discharge management according to national guidelines for the management of STIs using the syndromic approach were 119 (47.6%) and clinicians who mentioned the name of the drug, dosage, frequency, and duration correctly were 68 (27.2%). Clinicians who correctly mention the name of the drugs for vaginal discharge management were 95 (38%) and clinicians who mentioned the name of the drug, dosage, frequency, and duration correctly were 48 (19.2%). Out of all clinicians, clinicians who correctly mention the name of the drugs for genital ulcer management were 7 (2.8%) and clinicians who mentioned the name of the drug, dosage, frequency, and duration correctly were 5 (2%). From the total of 250 clinicians, clinicians who correctly mention the name of the drugs for pregnant women with vaginal discharge management were 4 (1.6%) and clinicians who mentioned the name of the drug, dosage, frequency, and duration correctly were also 4 (1.6%). And finally clinicians who correctly mention the name of the drugs in place of doxycycline for discharge management were 122 (48.8%) and clinicians who mentioned the name of the drug, dosage, frequency, and duration correctly were 75 (30%).

Overall knowledge of clinicians varied by syndrome type: it was highest for urethral discharge (27.2%) and lowest for pregnant women with vaginal discharge (1.6%).

When knowledge of clinicians was analyzed based on their training status on syndromic management of STIs, there was no significant difference among trained and untrained clinicians (Table 2).

### 3.4. Determinants of Knowledge of Clinicians

Further analysis to assess determinants of knowledge of urethral discharge shows that there were enormous variations on knowledge of urethral discharge among clinicians. Profession of clinicians and type of health facility they were working in were significant determinants of knowledge of urethral discharge. The other variables including training of clinicians on syndromic management of STIs were not statistically significant (Table 3).

Profession of clinicians was an important determinant on knowledge of urethral discharge. While 52.4% of health officers and 41.7% of medical doctors have knowledge on urethral discharge, only 22.4% of diploma nurses have knowledge on urethral discharge.

Health officers have knowledge of urethral discharge 4 times more likely than diploma nurses (AOR = 4.326; 95% CI = 2.019, 9.268). Likewise the odds of having knowledge of urethral discharge for medical Doctors were 8 times higher than diploma nurses (AOR = 7.788; 95% CI = 2.021, 30.006).

The other important determinant factor for knowledge of urethral discharge among clinicians was the type of health facility that clinicians are working in. 37.7% of clinicians working in health centers have knowledge of urethral discharge while only 14.3% of clinicians working in hospitals have knowledge of urethral discharge. Clinicians working in health centers have knowledge of urethral discharge 5 times more likely than clinicians working in hospitals (AOR = 0.194; 95% CI = 0.092, 0.412).

### 3.5. Practice of Clinicians

Of the total reviewed 120 cards of STI patients, only in 10 (8.3%) cards did the clinicians correctly follow the national guidelines and treat the patients correctly using the syndromic approach. Similarly, out of 26 mystery clients seen in 12 health facilities, only 5 (19.23%) were treated correctly according to the national guideline.

## 4. Discussions

This mixed method study assessed the knowledge and practice of clinicians regarding syndromic management of STIs in public health facilities of Gamo Gofa Zone, South Ethiopia. Accordingly the knowledge of clinician was only 27.2%. Only 8.3% follow the national guideline and few were trained on syndromic management of STIs.

The general compliance with the recommended national guideline of STI management is poor in this study. The prerequisites for implementation of the guideline were also poor. But, according to HIV/AIDS Prevention and Control Office, Ministry of Health, in order to treat STI patients, each clinician should follow national STI syndromic management guidelines [4]. At the same time to follow the guideline correctly, clinicians should have adequate training on syndromic management of STIs but in our study only 12.8% of clinicians were trained. This finding is higher than that of the finding from the study conducted in Windhoek, Namibia, among private general practitioners where none of them had specific training on syndromic management [12]. This could be attributed to the fact that our study was among clinicians working in public health facilities where government gives priorities whereas the study in Namibia was among clinicians working in private health facilities. However, the finding from this study is completely below the finding of the study conducted in South Africa where all clinicians working in 3 out of 4 clinics were trained on syndromic management of STIs [21]. This may be attributed to the fact that South Africa has been working the hardest to control STIs more.

In this study even if overall knowledge of clinicians varied by syndrome type it was all lowest; that is, the maximum knowledge the clinicians have was about treating urethral discharge correctly according to the national guideline that was 27.2%. This result is lower than that of the study conducted on KAP of general practitioners in Karachi, Pakistan, and among private general practitioners in Windhoek, Namibia, where the knowledge for urethral discharge was 55.3% and 56.5%, respectively [12, 13]. This might be because these studies only focused on knowledge of general practitioners while our study included lower level clinicians like diploma nurses also.

In this study from cards of STI patients reviewed, only in 8.3% cards did the clinicians correctly follow the national guidelines and treat the patients correctly using the syndromic approach. The other majority uses their experience to treat STI patients. This finding is almost consistent with the finding of the study conducted in Pakistan where most providers practiced personal empiricism, that is, following their own protocols for treatment with diverse medications, dosages, and treatment durations [14]. However, this finding is slightly lower than the finding of the study conducted in six countries in West Africa where effective treatment was given to 14.1% of patients in conformity with national syndromic STD management algorithms [15]. This might be attributable to the fact that this study covers the health facilities in six countries in West Africa which may include specialized health facilities while our study only included public health facilities in Gamo Gofa Zone of South Ethiopia.

Similarly in the study, of 26 mystery clients seen in 12 health facilities, only 5 (19.23%) were treated correctly according to the national guideline. When seen superficially, this finding seems to be a little bit higher than the finding of the study conducted in Pokhara, Nepal, where chemists and druggists treated patients correctly according to guideline in 10.8% of simulated client cases [22] but in fact chemists and druggists were not allowed to treat patients except referring them to doctors. So the finding of the current study shows low performance according to national guideline.

Concerning the limitation, the study was restricted to public health facilities in Gamo Gofa Zone only and the result does not represent private health facilities in the zone. The study measured the satisfaction status of mystery clients and not real clients which may have some difference.

## 5. Conclusions and Recommendations

In general the study indicated that the knowledge and practice of clinicians regarding syndromic management of STIs in public health facilities of Gamo Gofa Zone were poor.

Based on our results, efforts should be made to increase the knowledge of clinicians by providing training on syndromic management of STIs. After training, there needs to be supportive supervision to monitor and evaluate the actual performance of clinicians regarding syndromic management of STIs.

## Figures and Tables

**Table 1:** Sociodemographic characteristics of clinicians in public health facilities of Gamo Gofa Zone, Southern Ethiopia.

Variables	Number (*n* = 250)	Percentage (%)
*Profession*		
Diploma nurse	174	69.6
B.S. nurse	22	8.8
Health officer	42	16.8
Medical doctor	12	4.8
*Sex*		
Male	142	56.8
Female	108	43.2
*Age*		
Less than or equal to 29	141	56.4
30 to 39	70	28
40 to 49	24	9.6
50 to 59	13	5.2
60 and above	2	0.8
Mean ± SD (31.43 ± 8.31)		
*Work experience*		
Less than 1 year	5	2.0
1 to 5 years	115	46.0
6 to 10 years	71	28.4
Greater than 10 years	59	23.6
Median (6.0)		

**Table 2:** Clinicians knowledge of treatment for STI syndromes in public health facilities of Gamo Gofa Zone, Southern Ethiopia, 2015.

Knowledge of syndromes	Trained (32)	Untrained (218)	Total
*Has knowledge of urethral discharge *			
Yes	12 (37.5%)	56 (25.7%)	**68 (27.2%)**
No	20 (62.5%)	162 (74.3%)	182 (72.8%)
*Has knowledge of vaginal discharge *			
Yes	5 (15.6%)	43 (19.7%)	**48 (19.2%)**
No	27 (84.4%)	175 (80.3%)	202 (80.8%)
*Has knowledge of genital ulcer *			
Yes	1 (3.1%)	4 (1.8%)	**5 (2.0%)**
No	31 (96.9%)	214 (98.2%)	245 (98.0%)
*Has knowledge of pregnant women with vaginal discharge *			
Yes	2 (6.2%)	2 (0.9%)	**4 (1.6%)**
No	30 (93.8%)	216 (99.1%)	246 (98.4%)

Almost in all the above syndromes the knowledge for each syndrome among both trained and untrained clinicians was far below 50%: that shows that there was no significant difference in knowledge of all the above syndromes.

**Table 3:** Determinants of knowledge of urethral discharge in public health facilities of Gamo Gofa Zone, SNNPRS, Ethiopia, January 1 to February 28, 2015.

Determinants	*n* (%)	Have knowledge *n* (%)	Crude odds ratio (95% CI)	Adjusted odds ratio (95% CI)
*Profession of clinicians *				
Diploma nurse	174 (69.6)	39 (22.4%)	1	1
B.S. nurse	22 (8.8)	2 (9.1%)	0.346 (0.78, 1.546)	0.427 (0.092, 1.987)
Health officer	42 (16.8)	22 (52.4%)	3.808 (1.886, 7.688)^*∗*^	4.326 (2.019, 9.268)^*∗*^
Medical doctor	12 (4.8)	5 (41.7%)	2.473 (0.743, 8.223)	7.788 (2.021, 30.006)^*∗*^

*Type of health facility that clinicians are working in*				
Health center	138 (55.2%)	52 (37.7%)	1	1
Hospital	112 (44.8%)	16 (14.3%)	0.276 (0.147, 0.518)^*∗*^	0.194 (0.092, 0.412)^*∗*^

^*∗*^Significant results.
